# Hemodynamic influence of mild stenosis morphology in different coronary arteries: a computational fluid dynamic modelling study

**DOI:** 10.3389/fbioe.2024.1439846

**Published:** 2024-08-02

**Authors:** Xi Chen, Haoyao Cao, Yiming Li, Fei Chen, Yong Peng, Tinghui Zheng, Mao Chen

**Affiliations:** ^1^ Department of Mechanics and Engineering, College Architecture and Environment, Sichuan University, Chengdu, China; ^2^ Yibin Institute of Industrial Technology, Sichuan University, Yibin, China; ^3^ Department of Cardiology, West China Hospital, Sichuan University, Chengdu, China; ^4^ Med-X Center for Informatics, Sichuan University, Chengdu, China

**Keywords:** coronary arteriosclerosis, hemodynamics, computed tomographic, computational fluid dynamics, fluid mechanics

## Abstract

**Introduction:** Mild stenosis [degree of stenosis (DS) < 50%] is commonly labeled as nonobstructive lesion. Some lesions remain stable for several years, while others precipitate acute coronary syndromes (ACS) rapidly. The causes of ACS and the factors leading to diverse clinical outcomes remain unclear.

**Method:** This study aimed to investigate the hemodynamic influence of mild stenosis morphologies in different coronary arteries. The stenoses were modeled with different morphologies based on a healthy individual data. Computational fluid dynamics analysis was used to obtain hemodynamic characteristics, including flow waveforms, fractional flow reserve (FFR), flow streamlines, time-average wall shear stress (TAWSS), and oscillatory shear index (OSI).

**Results:** Numerical simulation indicated significant hemodynamic differences among different DS and locations. In the 20%–30% range, significant large, low-velocity vortexes resulted in low TAWSS (<4 dyne/cm^2^) around stenoses. In the 30%–50% range, high flow velocity due to lumen area reduction resulted in high TAWSS (>40 dyne/cm^2^), rapidly expanding the high TAWSS area (averagely increased by 0.46 cm^2^) in left main artery and left anterior descending artery (LAD), where high OSI areas remained extensive (>0.19 cm^2^).

**Discussion:** While mild stenosis does not pose any immediate ischemic risk due to a FFR > 0.95, 20%–50% stenosis requires attention and further subdivision based on location is essential. Rapid progression is a danger for lesions with 20%–30% DS near the stenoses and in the proximal LAD, while lesions with 30%–50% DS can cause plaque injury and rupture. These findings support clinical practice in early assessment, monitoring, and preventive treatment.

## 1 Introduction

Atherosclerosis is a chronic progressive vascular disease, typically characterized by the formation of plaques (known as coronary artery plaques) within arterial walls. Comprised of substances such as cholesterol, lipids, aggregated platelets, calcium salts, and fibrin, coronary artery plaques gradually lead to arterial stenosis and obstruction of blood flow (Gofman et al., 1950; Toussaint et al., 1996; Falk, 2006; Tavafi, 2013; Rafieian-Kopaei et al., 2014). Coronary artery disease (CAD) is the leading cause of death in the United States, accounting for approximately 610,000 deaths each year (one in four deaths), and approximately one in five deaths from CAD occurs in adults under the age of 65 years (Hajar, 2017).

The severity of stenosis is generally assessed using the Coronary Artery Disease Reporting And Data System (CAD-RADS) (Cury et al., 2016; Cury et al., 2022), which classifies the lesions into six grades (CAD-RAD 0-5) by the reduction of the lumen diameter. Patients classified as CAD-RADS >2 [degree of stenosis (DS) > 50%] are generally diagnosed as CAD and will receive treatments to avoid adverse events such as myocardial ischemia, hypoxia, and necrosis (Miller et al., 2008; Roffi et al., 2015; Cury et al., 2016; Nägele et al., 2020), while patients with CAD-RADS ≤ 2 are generally considered for preventive therapy or as non-atherosclerotic causes of chest pain (Cury et al., 2016). However, it is perplexing that some lesions remain stable for several years, yet some lesions precipitate acute coronary syndromes (ACS) in a short period of time (Ambrose et al., 1988; Glaser et al., 2005; Stone et al., 2011), which consists of coronary ischemia caused by coronary artery occlusion or partial occlusion, including plaque rupture or erosion (Sanchis-Gomar et al., 2016). The factors leading to varied clinical outcomes in similar non-obstructive lesions, and the causes triggering ACS from these lesions, remain unclear.

According to CAD-RADS, the clinical classification of the severity of CAD solely depends on the degree of stenosis, but clinical studies on severe coronary artery stenosis and renal artery stenosis have shown that the geometric morphology of stenosis also has an impact on adverse events (Xiong et al., 2021). In addition, stenosis location is also one of the influencing factors. For example, although the plaques with the same DS on the proximal and distal collateral arteries or on the left anterior descending artery (LAD) and non-LAD are described as the same CAD-RADS classification, the probability of risk events is different (Foldyna et al., 2018; Ramanathan et al., 2019). Up to now, for mild stenosis, there is no research on the relevant factors which may influence the hemodynamic environment. For non-obstructive lesions, should the morphology of the stenosis, such as the location, shape, be taken into consideration? Further, what combinations of stenosis morphological features warrant special attention in the clinical assessment?

Coronary Computed Tomography Angiography (CCTA) is a non-invasive method for visualizing CAD (Achenbach and Daniel, 2001; Budoff et al., 2008; Min et al., 2010). Over the past few decades, due to improvements in imaging quality, Computational Fluid Dynamics (CFD) analysis based on CCTA has been widely utilized to non-invasively calculate the hemodynamic variables. Previous research indicated that hemodynamic characteristics were closely related to CAD, as the CFD assessment enhanced the identification of high-risk plaques that subsequently caused ACS (Lee et al., 2019). The sensitivity and specificity of the computational results have been clinically validated (Kousera et al., 2013; Taylor et al., 2013; Kay et al., 2020).

Previous retrospective studies have largely focused on the evaluation of patients with severe coronary artery stenosis (Maurovich-Horvat et al., 2012; Johnson et al., 2014), with a lack of attention to early-stage atherosclerotic lesions. As our current understandings of these types of lesions are quite limited, it is necessary to assess the risk factors of mild stenosis based on hemodynamic analysis. In this study, we utilized CCTA images of a typical healthy individual to three-dimensionally reconstruct the original coronary artery model, implementing common stenosis characteristics on the proximal segmentations of different major coronary arteries for stenosis modeling, including stenosis length, eccentricity, and DS. CFD analysis was employed to obtain hemodynamic characteristics, including flow rate waveforms, Fractional Flow Reserve (FFR), Time-Averaged Wall Shear Stress (TAWSS), Oscillatory Shear Index (OSI), and streamlines. Our objective was to investigate the hemodynamic influence of various mild stenosis morphologies in different coronary arteries.

## 2 Materials and methods

This study retrospectively analyzed the CCTA lumen data of one individual, with the individual’s specific clinical records and CTA image data provided by West China Hospital of Sichuan University (Chengdu, Sichuan, China). As a typical healthy individual’s coronary artery for modeling, no plaque markers were found in the CTA images; the lumen size was uniform, the anatomical structure was normal, and the coronary artery path was typical.

This study was conducted in accordance with the principles of the Declaration of Helsinki and met relevant medical ethical requirements. The study was approved by the Ethics Review Committee of West China Hospital of Sichuan University.

### 2.1 Geometries

#### 2.1.1 Original model

**FIGURE 1:**
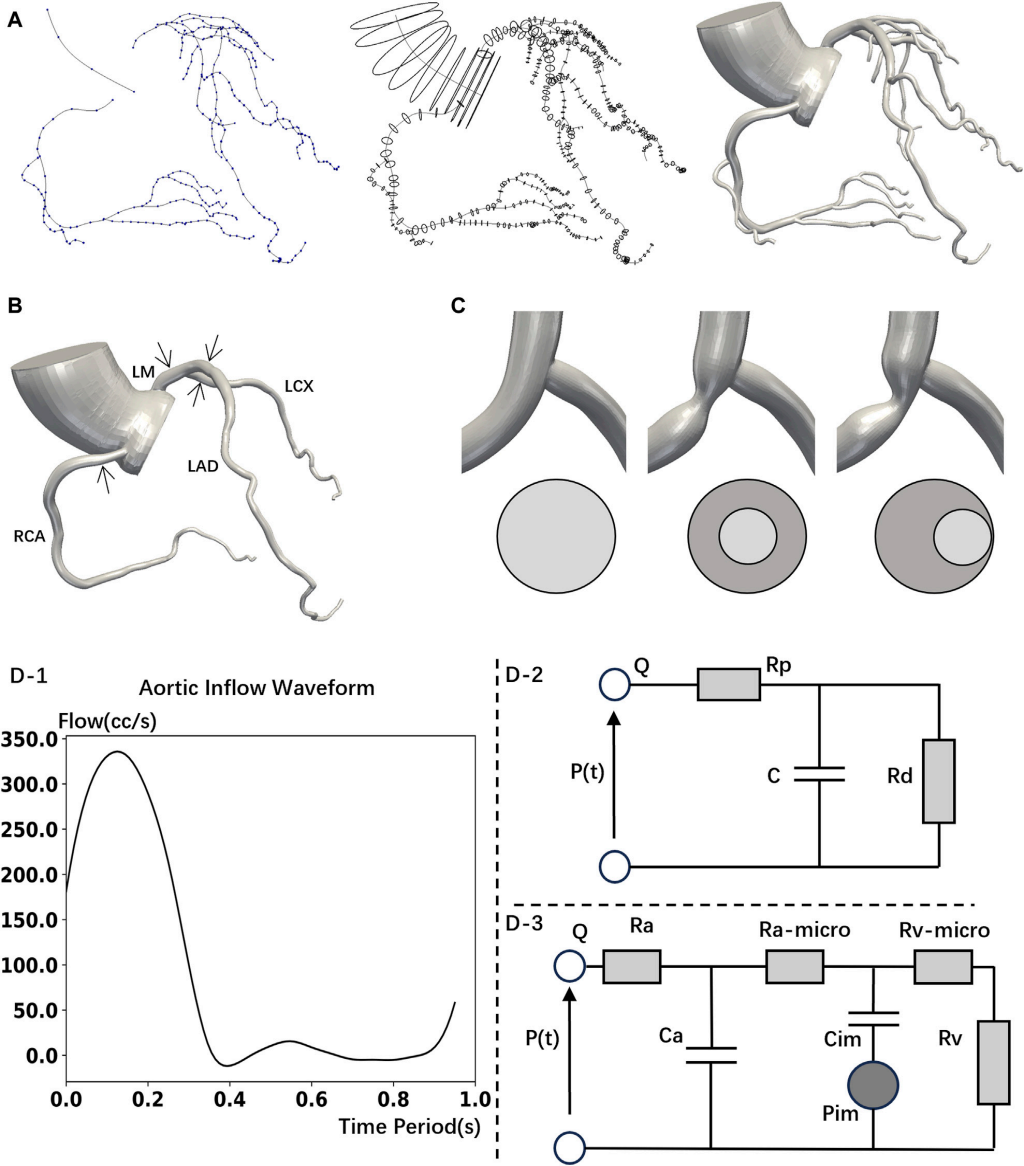
Model construction and boundary conditions set up. **(A)** 3D reconstructed mode is based on computed tomography images. Construct the centerlines of the vessels (left); segment the vessel perpendicular to the centerline (middle); 3D model (right). **(B)** Simplified model (No branch) with stenoses locations. **(C)** LAD Stenosis Model. Normal (left); Concentric (middle); Eccentric (right). **(D)**-1 Inlet flowrate wave. **(D)**-2 Wind Kessel RCR boundary conditions. **(D)-3** Lumped parameter network (LPN) coronary model.

The healthy coronary CCTA images were obtained using the second-generation computed tomography system (SOMATOM Definition CT, Siemens Medical Solutions, Forchheim, Germany), with a slice thickness of 0.6 mm. The open-source SimVascular software package was used to reconstruct a patient-specific three-dimensional model from the CTA data (Updegrove et al., 2017), including the coronary artery tree and aortic root, encompassing lumen centerline extraction, lumen contour segmentation, and three-dimensional modeling, as shown in Figure 1A.

Mesh construction uses unstructured tetrahedral elements with the TetGen library (Updegrove et al., 2017). According to the mesh independency verification, as shown in Table 1, the mesh size of the coronary artery was set to 0.05 mm, while the aorta was set to 0.5 mm. The boundary layer mesh consisted of four layers, with a height ratio of 0.6 and an initial height of 0.05 mm. The original model’s mesh consisted of 1,424,800 unstructured triangular faces.

**TABLE 1:** Mesh independency and stenosis parameters.

Mesh independency					
Values		Mean outlet flow rate		Mean pressure	Mean TAWSS
Percentage error (%)		0.21		0.8	1.84
Grid Independence index (%)		0.56		1.04	2.47
Stenosis parameters					
Case	Vessels	LM	LAD	LCX	RCA
	Length	0.7	0.62	0.5	0.63
Normal	Area	0.2	0.15	0.1	0.16
	Diameter	0.5	0.44	0.36	0.45
10% DS	Area	0.159	0.123	0.082	0.129
	Diameter	0.45	0.396	0.324	0.405
20% DS	Area	0.126	0.097	0.065	0.102
	Diameter	0.4	0.352	0.288	0.36
30% DS	Area	0.096	0.075	0.05	0.078
	Diameter	0.35	0.308	0.252	0.315
40% DS	Area	0.071	0.055	0.037	0.057
	Diameter	0.3	0.264	0.216	0.27
50% DS	Area	0.049	0.038	0.025	0.04
	Diameter	0.25	0.22	0.18	0.225
60% DS	Area	0.031	0.024	0.016	0.025
	Diameter	0.2	0.176	0.144	0.18
70% DS	Area	0.018	0.014	0.009	0.014
	Diameter	0.15	0.132	0.108	0.135

#### 2.1.2 Stenosed model

First, past research has shown that most coronary artery stenoses occur in the proximal parts of the arteries (Miller et al., 2009). In a coronary CTA survey of 418 subjects without prior history of coronary artery disease, W. Gregory Hundley et al. found that among subjects with single-vessel plaque, only 7% had distal plaque, while 75% had proximal plaque. Among subjects with a single location of plaque along a vessel, most had proximal plaque (69%); isolated distal-vessel plaque was rare (2%) (Grunfeld et al., 2010). Therefore, as a primarily study, the current research primarily focuses on the hemodynamic influence of stenoses located in proximal main arteries.

Second, past research often assumed the stenosis to be axially symmetrical (Deshpande et al., 1976; Ghalichi et al., 1998; Long et al., 2001). However, eccentric stenosis is also common in patients (Waller, 1989). Besides, eccentric stenosis will cause the flow to deviate from the centerline of the arteries (Timofeeva et al., 2022), and even lead to blood flow recirculation (Xie et al., 2018), which differs from the hemodynamics impact of concentric stenosis. Thus, to study the influence of stenosis eccentricity on hemodynamics, the shapes of stenoses were modelled with two types: concentric and eccentric. The lumen of the stenosis was a perfect circle, as shown in Figure 1C.

Third, although this study primarily focuses on mild stenoses (DS < 50%), severe stenoses with 60% DS and with 70% DS were also calculated to emphasize the hemodynamic differences between mild stenoses and severe stenoses.

In summary, to study the hemodynamic influence of stenoses on different main arteries, we modeled stenoses in the original model with different DS in proximal left main coronary artery (LM), LAD, left circumflex coronary artery (LCX) and right coronary artery (RCA). The plaque length was modeled using non-uniform rational B-splines, with length determined in previous studies (Plank et al., 2016; Newcombe et al., 2021), which is around 1.3 times the proximal vessel reference diameter for low-grade stenosis. Detailed parameters were given in Table 1.

To eliminate the specificity differences in calculations due to different branch locations among the patients, we removed the minor branches from the original model and retained only the main arteries (LM, LAD, LCX, RCA) to obtain the simplified models, as shown in Figure 1B.

The parameters for meshing remained consistent to original model, which were determined in previous studies on mesh independence analysis (Cao et al., 2021; Cao et al., 2023). The mesh count for the normal simplified model (original model without branches) amounted to 927,250 unstructured triangular faces.

### 2.2 CFD boundary conditions

In this study, the blood is assumed to be a laminar, transient, homogeneous Newtonian fluid. The governing equations are as follows, Eq. 1:

$$\rho \left(\frac{\partial \vec{u}}{\partial t}+\left(\vec{u}∙\nabla \right)\vec{u}\right)+\nabla p\;-\;\mu ∆\vec{u}=0$$ (1)

The incompressible condition, Eq. 2:

$$\nabla ∙\vec{u}=0$$ (2)

**FIGURE 2:**
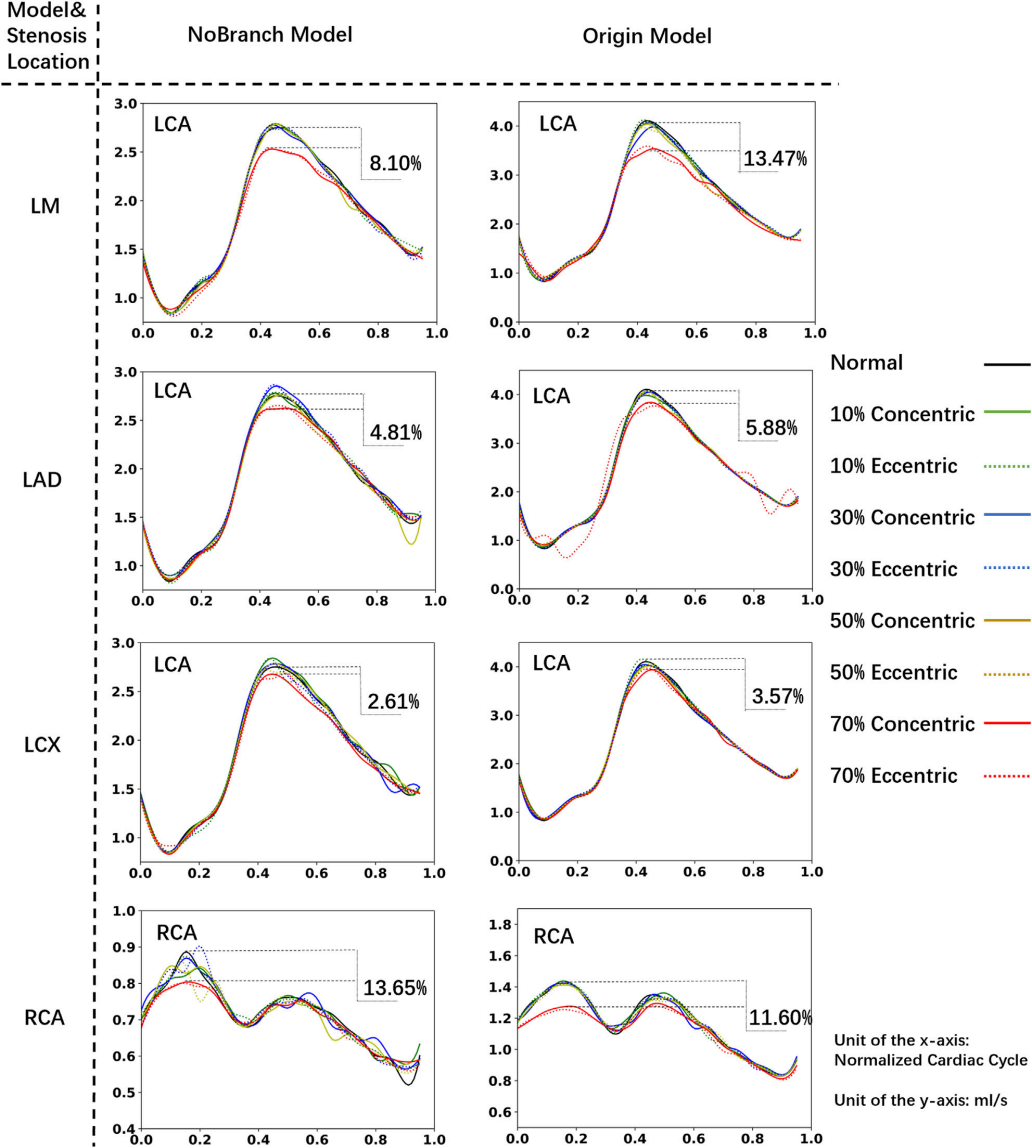
Coronary flow rate waveform. The xx% in the diagram represents the percentage reduction in peak flow rate caused by 70% stenosis compared to the normal model.

**FIGURE 3:**
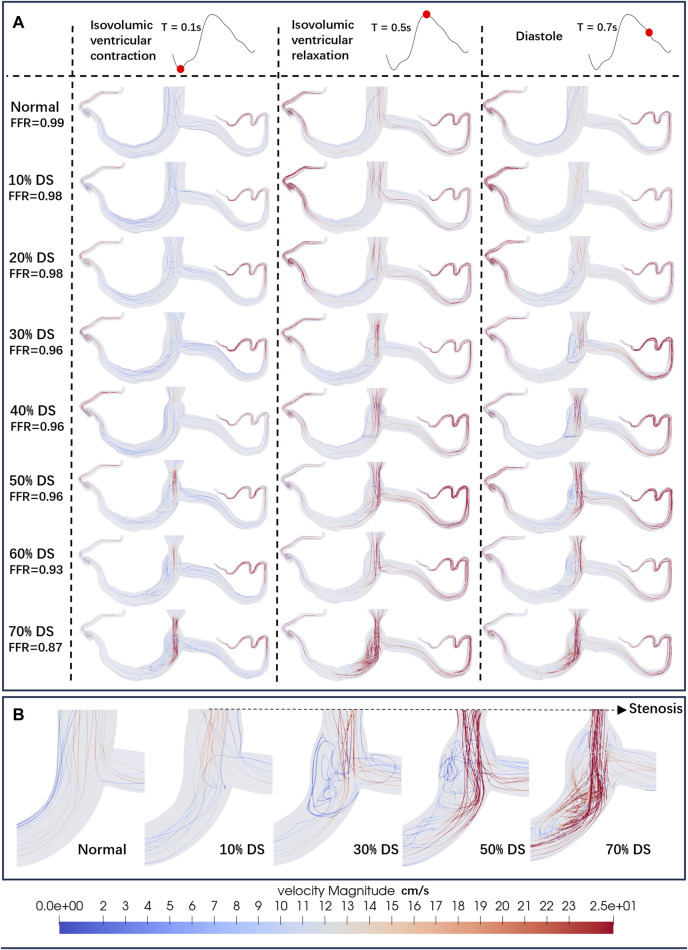
Streamlines of the blood flow. **(A)** Streamlines of the LM concentric stenosis model under different DS. The red dots on the left coronary flow waveforms indicate the acquisition time of the corresponding images below. **(B)** Detailed streamlines near the stenosis.

Where *u*, *p*, *ρ* and *µ* represent the fluid velocity vector, pressure, density (1.06 g/mL), and dynamic viscosity (0.04 dyne·s/cm^2^), respectively. All hemodynamic parameters were analyzed using the SimVascular software (Updegrove et al., 2017). At the aortic inlet, aortic outlet, and coronary artery outlet, the normal human blood flow waveform (Figure 1D-1), coronary artery boundary conditions (Figure 1D-2), and the lumped parameter network (LPN) coronary model (Figure 1D-3) were respectively utilized (Kim et al., 2009; Sankaran et al., 2012; Cao et al., 2023). The LPN model, being a simplified representation of the arterial system, primarily focuses on the overall behavior of the arterial system in terms of compliance and resistance (Olufsen, 2004; Westerhof et al., 2009). Previous studies have shown that mild stenosis, which causes only a slight narrowing of the artery, will not substantially impact the parameters of the system-wide LPN model (Zhou et al., 1999; Kim et al., 2010; Sankaran et al., 2012; Tran et al., 2017). Therefore, in this study which mainly focuses on mild stenosis, the parameters of the LPN model are determined by literature values for healthy individuals (Kim et al., 2009; Sankaran et al., 2012).

Boundary conditions, Eqs 3, 4:

$$R_{d}+R_{p}=P_{mean}/Q_{aorta}$$ (3)

$$R_{cor}:R_{aorta}=Q_{aorta\;}:Q_{cor}$$ (4)

R_p_ was the viscous resistance of the downstream arterial vessels, R_d_ represented the resistance of the capillaries and venous circulation, P_mean_ was the mean pressure, R_cor_ and Q_cor_ denoted the total resistance and cardiac output of the coronary arteries (mL/s), and R_aorta_ and Q_aorta_ represented the total resistance and cardiac output of the aorta, respectively. The resistances at each coronary artery outlet could be divided into R_a_ (arterial resistance), R_a-micro_ (microcirculatory resistance), R_v_ (venous resistance), C_a_ (microcirculatory compliance), C_im_ (myocardial compliance), and P_im_ (intra-myocardial pressure). These parameters were derived from healthy individuals according to the present study (Kim et al., 2009; Sankaran et al., 2012). The ratios were R_d_: R_p_ = 0.91: 0.09, C_a_: C_im_ = 0.11: 0.89. Vascular compliance (C) was 0.001 cm^5^/dyne.

Based on previous study (Cao et al., 2021), the simulation was set with 12 cardiac cycles, with each pulse cycle divided into 500 time steps. The simulation continued until the variation in the pressure field at the inlet and outlet did not exceed 1% compared to the previous cycle, and the data from the last cycle was chosen as the result.

In this study, the solution scheme involves implicit pressure coupling with a backflow stabilization coefficient of 0.2. The stabilized finite element method (FEM) formulation of the Navier-Stokes equations is discretized in time using a second-order backward differentiation formula. Spatial discretization is performed using a second-order finite element scheme.

### 2.3 Hemodynamic variables

Wall shear stress (WSS), which is proportional to blood viscosity and radial gradient, has been demonstrated to be a significant determining factor for endothelial function and gene phenotype (Malek et al., 1999).

The TAWSS can be used to evaluate the shear stress exerted on the wall by the pulsatile flow of cardiac circulation, which is defined as follows, Eq. 5:

$$TAWSS=\frac{1}{T}\int_{o}^{T}\left|WSS\right|dt$$ (5)

In the formula, T represents the cardiac cycle, and WSS is the vector of wall shear stress. Research has shown that abnormal TAWSS (<4 or >40 dyne/cm^2^) can lead to cell aggregation, platelet activation, and destructive remodeling mediated by inflammatory cells (Xiang et al., 2014). The low TAWSS (<4 dyne/cm^2^) that is commonly present in atherosclerosis-susceptible sites can stimulate the phenotypic characteristics leading to arterial atherosclerosis (Malek et al., 1999). Continuous quantitative coronary angiography has revealed that the local progression rate of arterial atherosclerosis in patients with CAD was highly correlated with low shear stress. This correlation held true even after controlling for systemic risk factors, such as circulating lipoprotein levels (Gibson et al., 1993). Correspondingly, high TAWSS (>40 dyne/cm^2^) is conducive to causing vascular lumen injury and unstable plaque formation (Samady et al., 2011; Gijsen et al., 2019).

Oscillatory shear stress (OSI) is a common indicator for evaluating the axial changes in WSS within a cardiac cycle, and it is defined as follows, Eq. 6:

$$OSI=0.5\times \left[1-\frac{\left|\int_{o}^{T}WSSdt\right|}{\int_{o}^{T}\left|WSS\right|dt}\right]$$ (6)

Where T is the period of the cardiac cycle, and WSS is the vector of wall shear stress. An high OSI (>0.2) indicates that the flow field is highly disturbed, which is associated with the formation of thrombosis (Xiang et al., 2014).

## 3 Results

### 3.1 Flow rate waveforms

Figure 2 reveals the influence of branches on the flow rate waveforms of the left coronary arteries (LCA) and the RCA. In various models, we observed that the flow rate of the LCA is low during systole and high during diastole, while the RCA’s waveform exhibits two characteristic peaks. Therefore, the physiologic behavior of coronary flow has been successfully captured: the blood flow of coronary arteries is impeded during systole and is increased during diastole promotes (Sengupta et al., 2012). Although mild stenosis has little effect on the flow rate in all the models, severe stenosis (70% DS) leads to a significant reduction in the flow rate and a visible deformation of the flow waveforms. Furthermore, severe stenosis in the proximal LM has the most substantial impact on the peak flow rate of the LCA. Compared to normal model, a 70% DS stenosis in the proximal LM causes 8.10% reduction of the LCA peak flow rate in the No-Branch Model (13.47% in the Origin Model). Besides, severe stenosis in the proximal RCA has the greatest effect on the first characteristic peak of the RCA flow waveforms, with a 13.65% reduction in the No-Branch Model (11.60% in the Origin Model).

According to the results, branch has the minimal impact (<1%) on flow rates and waveforms in cases of mild stenosis. At 70% DS, the difference between No-Branch model and Origin model in the impact on the ischemia proportion is within 5.37%. Namely, the impact of branches on flow is limited. The hemodynamic characteristics obtained using a simplified model without branches can be equivalent to those of a complex patient-specific model. Therefore, in subsequent analyses, we consistently used the model without branches.

### 3.2 Effect of lesion severity

Figure 3 shows the influence of different DS on the streamlines of the LM concentric stenosis model at 0.1s (isovolumic ventricular contraction), 0.5s (isovolumic ventricular relaxation) and 0.7s (diastole) in the cardiac cycle, as well as the FFR values. At the diastole phase (0.7s), chaotic flow become increasingly prominent as the DS increases. According to the CFD results, the FFR values in all mild stenosis models are higher than 0.95, consistent to the clinical observation of non-obstructive lesions (Cury et al., 2022). There are significant differences in the flow streamlines under various DS, specifically:

• 0% ≤ DS ≤ 20%: Characterized by a smooth stream tube, the flow was normal and laminar, with no obvious flow disorder or obstruction.
• 20% < DS ≤ 30%: Velocity of the flow begins to increase at the constriction of the blood vessel, and the flow starts to become chaotic. Large, low-speed vortexes appear near the stenosis.
• 30% < DS ≤ 50%: The velocity significantly accelerates around the stenosis and in the downstream of the flow. Vortex begins to spread downstream of the LAD. Small, high-speed vortexes appear from the stenosis to the LAD.
• 50% < DS ≤ 70%: The flow velocity further accelerates, leading to disordered flow. Simultaneously, large high-speed chaotic flow and small high-speed vortexes are present at and downstream of the stenosis.

**FIGURE 4:**
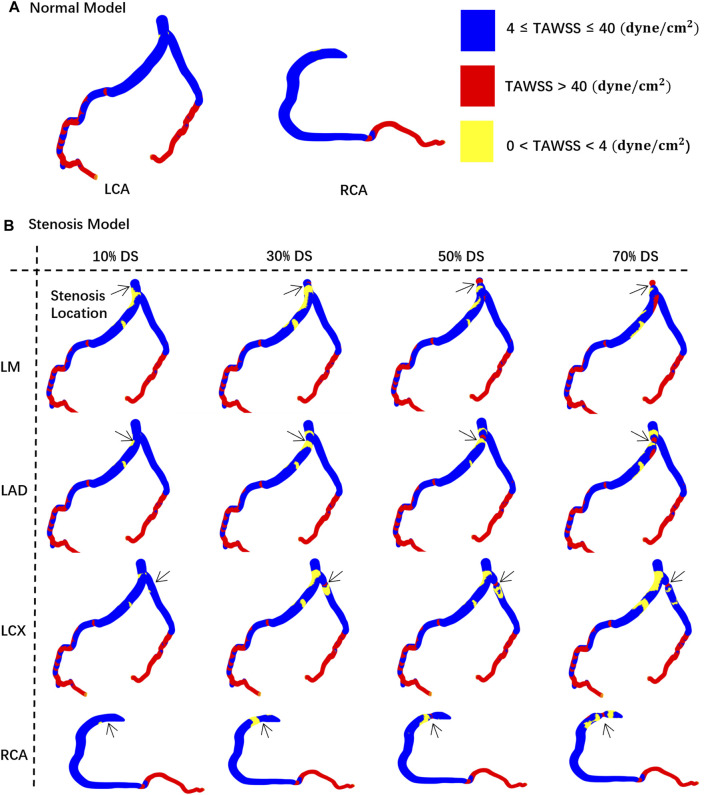
TAWSS distribution contour map on the vessel walls. The black arrow indicates the location of the stenosis. **(A)** Normal model. **(B)** Stenosis model.

Figure 4, using the concentric stenosis model as an example, shows the distribution of TAWSS abnormal regions (A: normal lumen, B: models at different DS).

**FIGURE 5:**
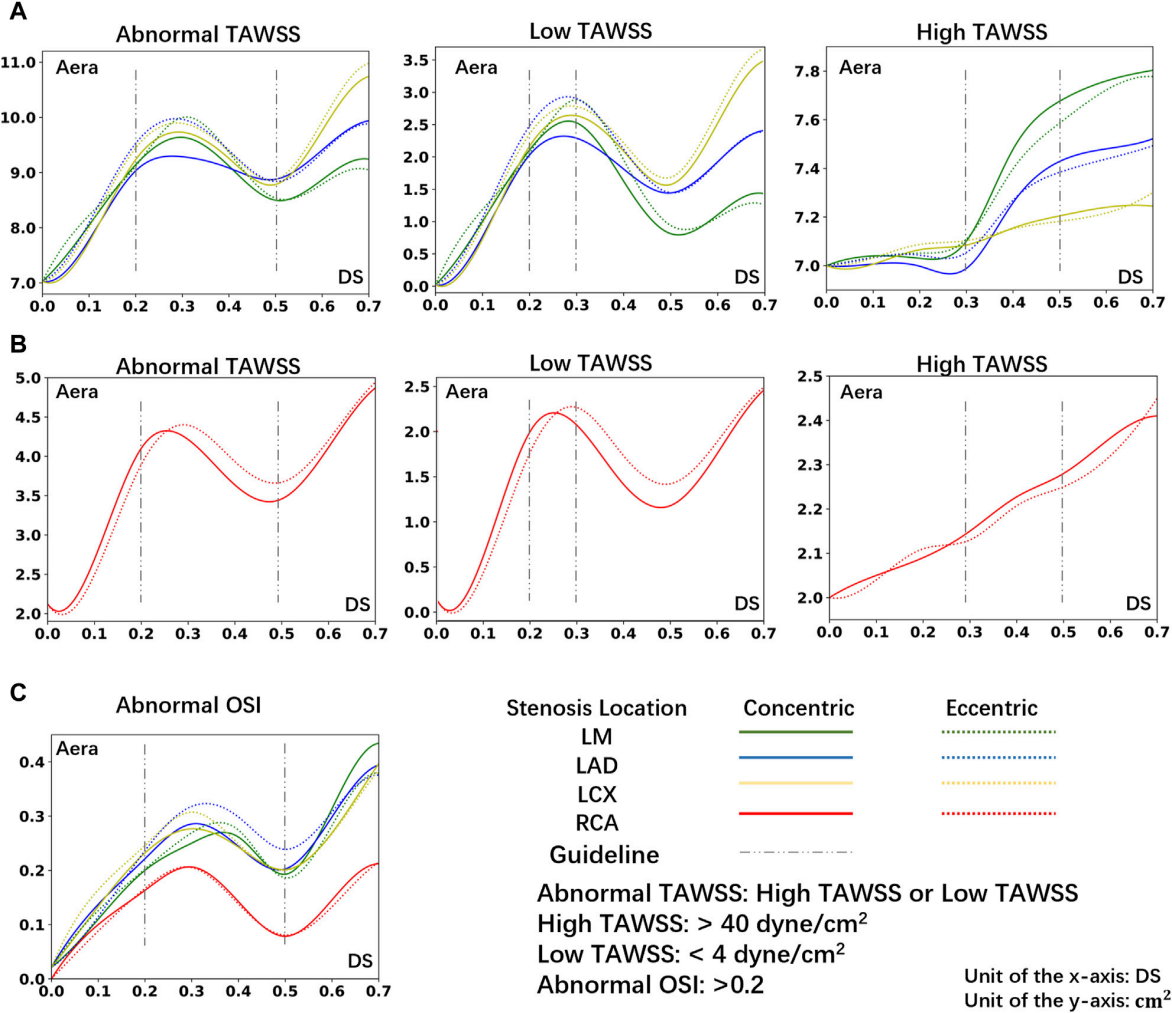
The area of abnormal flow regions (Abnormal TAWSS or Abnormal OSI) under different DS. **(A)** The area of abnormal TAWSS in LCA under different DS. **(B)** The area of abnormal TAWSS in RCA under different DS. **(C)** The area of abnormal OSI in LCA or RCA under different DS.

Figure 5 illustrates the relationship between the area of hemodynamic abnormality regions on the four main arteries, and morphological parameters, including the corresponding location, eccentricity, and DS. Each curve in the graph is composed of eight anchor points (DS = 0, 10%, 20%, 30%, 40%, 50%, 60%, 70%), and B-spline interpolation is used for smooth plotting.

Consistent with prior research (Stroud et al., 2000), in the instance of severe stenosis (DS = 60%, 70%), a large region of hemodynamics abnormalities is observed in the downstream area. In the LCA, the areas with OSI > 0.2 due to severe stenosis are all greater than 0.26 cm^2^. In the RCA, these areas each exceed 0.13 cm^2^. Meanwhile, in the LCA, areas with TAWSS > 40 dyne/cm^2^ or <4 dyne/cm^2^ are all greater than 8.8 cm^2^. In the RCA, these areas each exceed 4.1 cm^2^. However, the size of abnormal area is not linearly proportional to the DS. In cases of mild stenosis, the size of hemodynamics abnormal area initially increases with the DS, reaching a local peak before gradually declining, and then rapidly enlarges when DS > 50%. The DS interval between 20% and 50% warrants special consideration:

• 20%–30%: Due to the emergence of large, low-velocity vortexes (Figure 3), the low TAWSS areas have appeared near the stenosis in the model (Figure 4), which has led to the occurrence of local peak intervals of abnormal TAWSS (Figure 5).
• 30%–50%: Owing to a marked increase in downstream flow velocity near the stenosis (Figure 3), high TAWSS regions appeared around the stenosis (Figure 4), resulting in a rapid expansion of the high TAWSS area (increase by 0.46 cm^2^ on average) in the LM and LAD (Figure 5). Simultaneously, the area of the high OSI regions has continuously maintained a relatively high level, which is all above 0.19 cm^2^.

### 3.3 Effect of lesion location

In models of different DS, the size of hemodynamics abnormal area in stenotic LCA (Figure 5A) is always much larger than that in stenotic RCA model (Figure 5B). For mild stenoses (DS = 10%, 20%, 30%, 40%, 50%), compared to the LCA, the size of TAWSS and OSI abnormal areas of stenotic RCA model are reduced by 59.44% and 39.78% on average, respectively. Considering the difference in surface area between the RCA and LCA, the normalized size of TAWSS and OSI abnormal areas of the stenotic LCA model is still greater than that of the stenotic RCA model (37.70% and 7.54%). Among them, the low TAWSS area in the LCA is concentrated in the proximal segments of the LAD, while the low TAWSS area in the RCA is concentrated near the stenosis (Figure 4).

Within the DS of 30%–50%, the size of high TAWSS area rises more quickly in the stenotic LM and LAD models (average Δ = 0.46 cm^2^, Figure 5). However, in the models of stenosis in the LCX and RCA, the area of high TAWSS regions rises more slowly when the DS increases from 30% to 50% (average Δ = 0.11 cm^2^, Figure 5).

### 3.4 Effect of lesion eccentricity

For mild stenoses (DS = 10%, 20%, 30%, 40%, 50%), the hemodynamics abnormal area caused by eccentric stenosis located in the LCA is consistently larger than that caused by concentric stenosis. In the LCA, compared to concentric stenosis, the size of TAWSS abnormal area caused by eccentric stenosis is on average higher by 2.61%, while the OSI abnormal area is on average higher by 7.20%. However, no significant difference in the hemodynamics effect is observed between concentric and eccentric stenosis in the RCA (Figure 5).

### 3.5 Multiple study

Table 2 enumerates the effects on flow of four lesions with different morphological parameters under a DS of 40%. From the table, eccentric stenosis located in the LM has a far greater adverse effect on hemodynamics characteristics than concentric stenosis with the same DS located in the RCA.

**TABLE 2:** Influence of multiple morphologies on the 40% DS model flow.

Parameters		Abnormal TAWSS area (cm^2^)			High OSI area (cm^2^)
Location	Eccentricity	Total	Low*	High**	OSI > 0.2
LM	Eccentric	9.37	1.97	7.40	0.27
LM	Concentric	9.11	1.64	7.48	0.26
RCA	Eccentric	3.93	1.72	2.21	0.14
RCA	Concentric	3.64	1.41	2.23	0.14

Note: *TAWSS < 4 dyne/cm^2^; **TAWSS > 40 dyne/cm^2^.

## 4 Discussion

Although stenoses with DS < 50% are typically considered to be non-obstructive lesions according to the CAD-RADS (Cury et al., 2016), mild stenosis may still lead to future ACS(Ambrose et al., 1988; Glaser et al., 2005; Stone et al., 2011). Moreover, CAD-RADS simply classifies patients into different grades solely by DS, with potential oversight of lesion morphologies and locations, which might be the reasons for the clinical underestimation of non-obstructive lesions (Foldyna et al., 2018). It is well acknowledged that the hemodynamic environment plays a crucial role in the stenosis initiation/progression, plaque rupture/erosion, vascular remodeling, and even coronary functional changes that occur in eventual myocardial ischemic events (Feldman and Stone, 2000; Cecchi et al., 2011). Accordingly, this study, based on the CTA images of a typical healthy individual, investigated the influence of mild stenoses with various locations, DS, and eccentricity on myocardial perfusion and hemodynamics characteristics. The results indicate that for mild stenosis, although the FFR values and flow rate are within the normal range, mild stenosis with specific morphologies still presents hemodynamic abnormalities, which deserves particular attention. In addition, despite that minor branches have a minimal influence on myocardial perfusion capability (<5.37%) and influence of lesions’ eccentricity on the hemodynamic abnormality is limited (<7.20%), the DS and location have a more significant impact on the hemodynamic characteristics. Overall, the stenosis located in LCA with a 20%–50% DS warrants special consideration.

Firstly, FFR is currently the gold standard diagnostic index for invasive assessment of the severity of coronary artery stenosis, which is derived from the ratio of the distal pressure divided by the proximal pressure or aortic pressure under maximal hyperemic conditions during invasive coronary angiography (Pijls et al., 1993). Current consensus suggests that an FFR value of less than 0.8 may induce myocardial ischemia (Tu et al., 2020). Consistent with clinical reports, this study revealed that FFR values in mild stenosis models are all greater than 0.95, which indeed shows there may be no risk of immediate ischemia. However, this CFD study identified numerous regions of abnormal hemodynamics including abnormal low and high TAWSS, high OSI in these DS < 50% models, such as proximal segments of LAD and regions near stenosis (Figure 4). It has been suggested abnormal low TAWSS (<4 dyne/cm^2^) which is commonly present in atherosclerosis-susceptible sites can stimulate the phenotypic characteristics leading to arterial atherosclerosis (Malek et al., 1999), abnormal high TAWSS (>40 dyne/cm^2^) is conducive to causing vascular lumen injury and unstable plaque formation (Samady et al., 2011; Gijsen et al., 2019) and high OSI (>0.2) is associated with the formation of thrombosis (Xiang et al., 2014). Thus, the abnormal hemodynamics presented in the mild stenosis might trigger the onset of ACS, including rapid progression, rupture, and erosion of the lesion (Overbaugh, 2009).

Secondly, previous research based on clinical follow-up study indicated that the growth patterns and progression speed of atherosclerosis differ among the different coronary arteries (Bax et al., 2022), as the LAD’s proximal segments exhibits an increased susceptibility to atherosclerosis (White et al., 1950; Montenegro and Eggen, 1968). Focusing on the non-obstructive lesions, this current study showed that stenoses situated in the LCA consistently result in larger abnormal hemodynamics regions compared to those located in the RCA, as the size of TAWSS abnormal area caused by RCA mild stenosis is on average 59.44% lower than the LCA. Besides, it is observed that the abnormal low TAWSS regions of DS within 20%–30% are primarily concentrated in the proximal segments of the LAD and LM (Figure 4), which aligns with the clinical susceptible locations for atherosclerosis. Moreover, Kolodgie et al. found in autopsies of 113 patients who succumbed to sudden death due to CAD that thin-cap fibroatheroma lesions were primarily located in the proximal and mid segments of the LAD and the proximal segment of the LCX (60%), followed by the proximal and mid segments of the RCA (20%) (Kolodgie et al., 2001; Wykrzykowska et al., 2012). In this study, it is discovered a rapid increase in abnormal high TAWSS region area in the LM and LAD in the 30%–50% DS interval (Figure 5B). Finally, at an identical DS of 40%, as shown in multiple study, the area of high TAWSS and OSI caused by stenosis in the LM is on average 3.35 times and 1.89 times greater than that in the RCA. That is, for non-obstructive stenoses, the locations of stenoses exert a considerable influence on the size and distribution of hemodynamics abnormal regions, as a stenosis located in LCA may have a higher risk of progression, development, and even rupture than another stenosis of the same degree located in RCA. Thus, the various locations of lesions may be a crucial factor causing different clinical outcomes.

Finally, all stenoses with DS < 50% are clinically classified as non-obstructive lesions currently. However, it is worth to note that the current study shows that the size of hemodynamics abnormal area is not simply linearly proportional to the DS. To be in detail, within the DS of 20%–30%, the appearance of large-scale, low-speed vortexes lead to the emergence of hemodynamics abnormal bands dominated by low TAWSS (<4 dyne/cm^2^) areas near the stenosis. In addition, within the DS of 30%–50%, the noticeable acceleration of flow speed near the stenosis leads to the emergence of high TAWSS, resulting in a rapid increase in the size of the high TAWSS (>40 dyne/cm^2^) area and a large area of high OSI (>0.2). As stated above, mild stenosis (DS < 50%) should be further subdivided from a hemodynamic perspective. Patients of DS within 20%–30% may have a risk of rapid progression of stenosis (Kaski et al., 1995), which requires intensified follow-up to monitor potential rapid narrowing or expansion of the plaque. Conversely, patients of DS within 30%–50% may be susceptible to experiencing plaque injury or rupture (Chang et al., 2018), necessitating further clinical evaluation such as additional check of the LAD proximal segments, medication requirements (the statin, the aspirin, etc.) to prevent potential ACS. In summary, although stenoses with DS <50% are all classified as non-obstructive lesions, patients in different DS interval (20%–30% or 30%–50%) face different risks, which may be another key factor leading to different clinical outcomes.

There were some limitations in the present study. First, the values of the parameters in the boundary conditions are taken from healthy individuals. Although that localized, single stenosis has little impact on the boundary conditions like overall waveform of the aorta, for cases of DS > 50%, the boundary conditions might slightly differ. Second, the article used a CTA image modeling analysis from a healthy individual. Although this is sufficient to elucidate the relationship between anatomy and the hemodynamics of blood flow, it does not cover all clinical cases. In real clinical practice, there might be various results due to the uniqueness of the patient’s specific coronary arteries. In addition, we used models without branches rather than the original model for our study. Although in Section 3.1, we noted that this simplification is effective, the original model is closer to the real situation in vessels. The presence of small branches helps to lower the resistance in the main vessels and distribute the blood flow, which helps to smooth the blood flow and increase the flow rate, as shown in Figure 2. What’s more, this study used the Newtonian assumption rather than a non-Newtonian model. Although existing studies have shown that assumptions on blood rheology have negligible impact on CFD hemodynamic quantities linked to atherosclerotic coronary artery disease (De Nisco et al., 2023), we acknowledge that a non-Newtonian flow model could better simulate the actual blood flow conditions. At last, due to time constraints, we could only apply hemodynamics study on some important lesion morphologies arrangements and combinations, without considerations of many other variables like branch location, stenosis shape, etc. Thus, our future research will concentrate on the following areas: 1) Does the shape of the stenosis and branch location affect the area and distribution of the hemodynamics abnormal region? 2) Do individual differences in a patient’s medical history (such as diabetes, hypertension) have a significant effect on coronary flow?

## 5 Conclusion

The study revealed that aligning with the current standardize reporting system, plaques with a DS < 50% have no immediate risk of ischemic in terms of FFR. However, although lesions with a DS of 20%–50% are non-obstructive, they can still cause hemodynamic abnormalities, which may be a potential cause of ACS and merits special attention, especially the lesions located in LCA. From the perspective of hemodynamics, the lesion location and DS may be possible reasons for the different clinical outcomes.

In addition, this study suggest that mild stenosis may should be further subdivided: those within of 20%–30% range are prone to experiencing rapid progression of plaque near the stenosis and in the proximal segment of LAD, and patients with a 30%–50% stenosis located in LM or LAD, are susceptible populations to plaque injury and rupture.

The findings of this study assist clinical practice in early assessment, providing strategies for early monitoring and supportive treatment, and may also contribute to further clarification of clinical outcomes for patients.

## Data Availability

The original contributions presented in the study are included in the article/Supplementary Material, further inquiries can be directed to the corresponding authors.
